# Fluorescent Neoglycoprotein Gold Nanoclusters: Synthesis and Applications in Plant Lectin Sensing and Cell Imaging

**DOI:** 10.1186/s11671-018-2772-2

**Published:** 2018-11-12

**Authors:** Katarzyna Alicja Brzezicka, Sonia Serna, Niels Christian Reichardt

**Affiliations:** 1 Glycotechnology Laboratory, CIC biomaGUNE, Paseo Miramon 182, 20014 San Sebastian, Spain; 2 Departments of Molecular Medicine and Microbiology and Immunology, The Scripps Research Institute, La Jolla, CA 92037 USA; 3 CIBER-BBN, Paseo Miramon 182, 20014 San Sebastian, Spain

**Keywords:** Neoglycoproteins, Carbohydrate-protein interactions, Gold nanoclusters, Targeting, Lectin sensing, Dendritic cells

## Abstract

Carbohydrate-protein interactions mediate fundamental biological processes, such as fertilization, cell signaling, or host-pathogen communication. However, because of the enormous complexity of glycan recognition events, new tools enabling their analysis or applications emerge in recent years. Here, we describe the first preparation of neoglycoprotein functionalized fluorescent gold nanoclusters, containing a biantennary N-glycan G0 as targeting molecule, ovalbumin as carrier/model antigen, and a fluorescent gold core as imaging probe (G0-OVA-AuNCs). Subsequently, we demonstrate the utility of generated G0-OVA-AuNCs for specific sensing of plant lectins and in vitro imaging of dendritic cells.

**Electronic supplementary material:**

The online version of this article (10.1186/s11671-018-2772-2) contains supplementary material, which is available to authorized users.

## Introduction

Gold nanoclusters (AuNCs) formed by ten to hundred atoms of gold have gained the attention of scientific community due to their attractive chemical and physical properties [1]. Smaller than 3 nm, gold nanoclusters approach the Fermi wavelength of electrons giving rise to size-dependent fluorescence emission and offering opportunities as sensing and imaging probes for in vitro and in vivo applications [2–4]. Current fluorescent assays mostly involve organic dyes, such as rhodamine or fluorescein, or less commonly quantum dots [5–7]. However, due to the low photochemical stability, pH-sensitive fluorescence, or poor water solubility of some organic dyes and toxicity of quantum dots, their use can be compromised [8]. In this context, gold nanoclusters can be considered alternative ultra-small fluorophores, lacking above-mentioned limitations. Furthermore, AuNCs are characterized by a large Stokes shift and fluorescence emission wavelength from the red visible to the near-infrared (IR) region that is highly favorable in bioimaging because it overlaps the tissue transparency window [8–10].

Protein-assisted synthesis of gold nanoclusters was first reported in 2009 using bovine serum albumin (BSA) [11] and since then, water-soluble protein-protected nanoclusters have become an emerging trend in nanoscience [9, 12, 13]. Far less attention has been given to glycoproteins [14] for the preparation of AuNCs and we are not aware of any reports describing AuNCs formation from synthetic neoglycoproteins as scaffolds. In general, glycosylation modulates the physicochemical properties of glycoproteins, e.g., folding, circulatory life-time, or stability. It also affects important biological functions of protein, such as receptor-ligand recognition. Thus, the generation of synthetic neoglycoproteins from proteins, by the chemical attachment of carefully designed and characterized carbohydrates [15], can equip them with new functional properties for biological applications. As carbohydrates participate in a great number of different biological processes through the interaction with carbohydrate binding proteins [16, 17], we envisage neoglycoprotein gold nanoclusters as novel probes to study and exploit carbohydrate recognition events both in vitro and in vivo [18]. In this regard, the presence of multiple glycan copies on the protein surface provides a multivalent presentation of carbohydrates with the subsequent enhancement of binding affinity.

Here, we explore the use of self-fluorescent neoglycoprotein functionalized AuNCs as sensing probes for plant lectins and as targeting reagents of lectin receptors for in vitro imaging of dendritic cells (DCs). Lectins are carbohydrate binding proteins that assist in different biological recognition phenomena. In higher plants, for example, lectins prevent from plant-eating organisms by recognition and agglutination of foreign glycoproteins, inhibiting their growth and multiplication [19]. In mammalian DCs, on the other hand, C-type lectin receptors (CLRs) express on the cell surface, play a major role in pathogen recognition [20]. Glycan-decorated antigens are recognized by specific CLRs, to be further endocytosed, processed, and eventually presented to T cells inducing specific immune responses. In our previous study, we showed [21] that the functionalization of a model antigen, ovalbumin (OVA), with a synthetic biantennary GlcNAc terminating N-glycan G0 enhances targeting to DCs and subsequent antigen uptake and presentation. We postulated that above-mentioned phenomenon is initiated by the interaction of G0 glycan and endocytic C-type lectin receptors expressed on the surface of DCs. Consequently, we believe that fluorescent and multivalent G0-OVA gold nanoclusters could become an alternative to fluorescently labeled G0-OVA applied in our previous study and could be used as a novel tool for DC visualization. Moreover, glycan-mediated targeting of DCs which enables initiation of strong T cell immune responses could be used to enhance the efficacy of vaccine candidates [22–24]. In this initial study, we will present the synthesis of gold nanoclusters starting from neoglycoproteins and the evaluation of the functionality and accessibility of G0 glycans using lectin agglutination experiments. Finally, we will demonstrate the potential of fluorescent gold nanoclusters for dendritic cells imaging.

## Results and Discussion

**Fig. 1:**
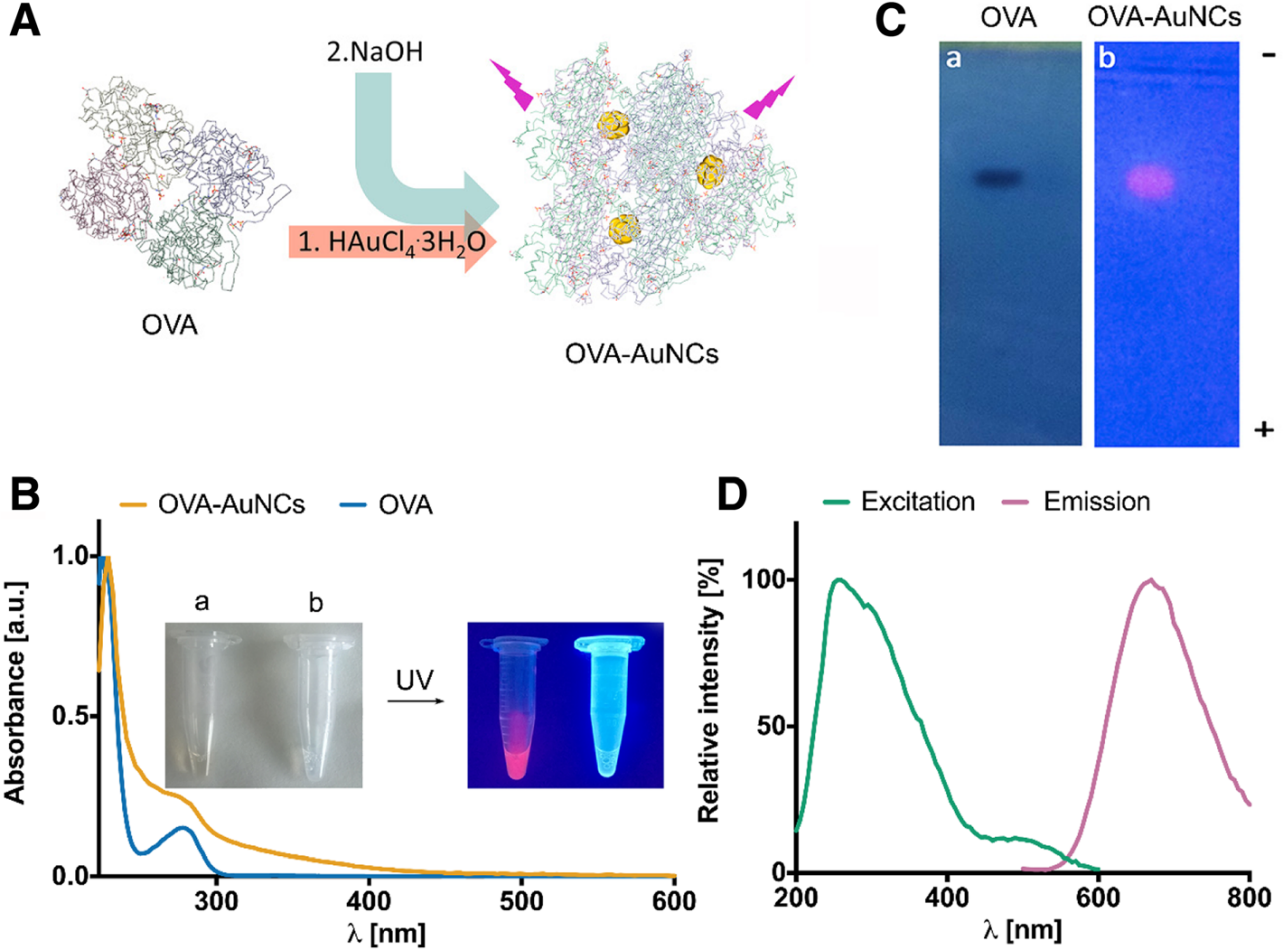
**a** Schematic representation of OVA-AuNCs synthesis. **b** UV-visible spectra of OVA (blue line) and OVA-AuNCs (orange line). Insert: images of OVA-AuNCs (**a**) and OVA (**b**) under visible light illumination (left) and under UV light illumination (365 nm) (right). **c** Agarose gel electrophoresis of OVA (stained with Coomassie Blue G-250) and OVA-AuNCs (under UV light illumination). **d** Fluorescence excitation (green line) and emission (pink line) spectra of OVA-AuNCs

We optimized the synthesis of ovalbumin gold nanoclusters decorated with the G0 glycan (G0-OVA-AuNCs) based on previous reports employing unconjugated OVA protein [25, 26]. Protein-protected AuNCs were prepared by the addition of gold tetrachloroauric (III) acid (HAuCl_4_^.^3H_2_O) to a protein solution, followed by 1 M aqueous solution of sodium hydroxide. An increase of the reaction pH enhances the reduction potential of tryptophan and tyrosine residues present in OVA (Fig. 1a) [14]. The protein scaffold acts as both reductive and stabilizing reagent, entrapping the small gold cluster inside the protein structure and isolating it from the environment. A previous protocol for the synthesis of fluorescent OVA-AuNCs required a very highly concentrated OVA solution (up to 65 mg mL^−1^) [25]. To limit the use of valuable OVA neoglycoconjugates for the preparation of AuNCs, we determined the minimum amount of unconjugated OVA that could efficiently produce OVA-AuNCs. We found that an OVA concentration of 15 mg mL^−1^ was sufficient to produce clusters with strong fluorescence emission (Additional file 1: Figure S1). The formation of OVA-AuNCs was significantly accelerated by microwave irradiation reducing the reaction time from 18 h to 6 min [25]. In addition, microwave irradiation provided homogeneous heating to the solution favoring the formation of uniform and monodisperse clusters [1]. OVA-AuNCs prepared in this manner exhibit a pale brown color and emit a strong red fluorescence under UV light illumination (Fig. 1b). The UV-Vis spectrum of OVA-AuNCs lacks the absorbance corresponding to a localized surface plasmon resonance band suggesting the absence of gold nanoparticles larger than 5 nm [27] which was further confirmed by transmission electron microscopy (TEM). We measured an average diameter of the OVA-AuNCs gold core in a range of 1.9 ± 0.7 nm (Additional file 1: Figure S2). Finally, OVA-AuNCs average hydrodynamic size of 8.7 nm ± 2.5 nm was assigned by dynamic light scattering (Additional file 1: Figure S2). Its similar size range to OVA (6.5–7 nm diameter [28]) suggests a presence of a single protein molecule per gold core. Further analysis of OVA-AuNCs by agarose gel electrophoresis showed a similar mobility for OVA and OVA-AuNCs toward a positive electrode that further confirms the similar size for both species and their negative charge at neutral pH (Fig. 1c). The fluorescence emission spectrum of OVA-AuNCs shows a maximum emission peak at λ 670 nm upon excitation at 350 nm (Fig. 1d). The excitation spectra of AuNCs is broad and the Stokes shift large (above 200 nm) which is ideal for spectral multicolor detection applications in a presence of another fluorescent probe (multiplexing) characterized by similar excitation wavelength, such as blue emitting Alexa Fluor® 405 dye [26]. The quantum yield (QY) of OVA-AuNCs was calculated to be ≈ 4% when fluorescein in 0.1 M NaOH (QY = ≈ 92%) was used as reference standard [29]. The oxidation state of gold core was assigned to a mixture of Au(I) and Au(0) species based on X-ray photoelectron spectroscopy (XPS) measurements (Additional file 1: Figure S3) [11, 30]. Study of the protein secondary structure circular dichroism (CD) revealed a random coil organization suggesting a loss of the native fold for OVA in AuNCs probably due to harsh alkaline conditions during synthesis (Additional file 1: Figure S4) [31]. Finally, we have also observed an extraordinary stability of OVA-AuNCs within a broad range of pH (3–11) as well as in a solution containing fetal bovine serum (FBS), the most commonly used serum-supplement for in vitro cell culture (Additional file 1: Figure S5). This feature of OVA gold nanoclusters highlights their utility for in vitro and in vivo bioassays and opens up exciting new applications. For instance, the pH-insensitive fluorescence emission of OVA-AuNCs could permit efficient particle tracking inside the cell without loss of signal even inside the endosomal compartments, characterized by slightly acidic pH [32, 33]. In contrary, under these conditions, the use of certain fluorescein conjugates can be compromised, as the maximal fluorescence emission of these dyes is achieved in the basic pH. OVA-AuNCs also showed an excellent solubility both in water and in cell growth medium. Complete solubilization of OVA-AuNCs was observed up to 40 mg/mL concentration both in water and in cell growth medium. (Additional file 1: Figure S6). Fluorescence emission spectra at different OVA-AuNCs dilutions were measured and probed to be stable under incubation at 37 °C up to 24 h.

**Fig. 2:**
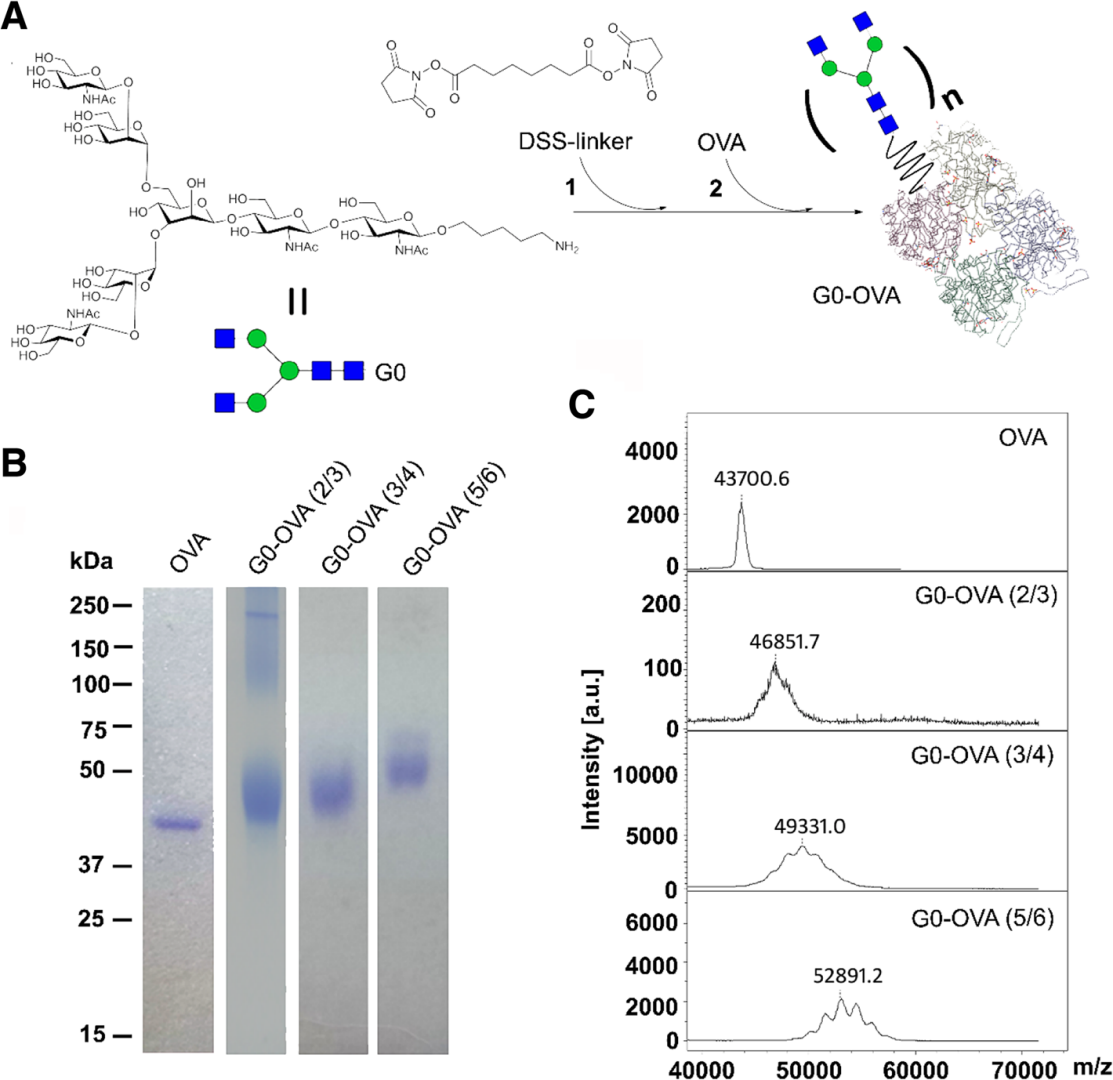
**a** Synthesis of G0-OVA neoglycoconjugates (*n* = valency). **b** SDS-PAGE gel electrophoresis of OVA and G0-OVA neoglycoproteins containing different valencies of N-glycan G0. **c** MALDI-TOF mass spectra of OVA and G0-OVA neoglycoproteins containing different valencies of N-glycan G0

For the synthesis of G0-OVA neoglycoprotein-protected AuNCs, we initially prepared G0 functionalized OVA neoglycoproteins. Biantennary N-glycan G0 equipped with a C5 amino linker was synthesized as previously described [34] and the conjugation with OVA was achieved employing disuccinimidyl suberate ester (DSS) as crosslinking reagent (Fig. 2a) [21, 35]. In brief, the conjugation is performed in a two-step reaction: N-glycan G0 is functionalized with a 13-fold excess of DSS linker and then coupled to free amino groups on OVA. By controlling the glycan/protein ratio, we could effectively adjust the degree of OVA substitution (see SI). The successful formation of neoglycoproteins was verified by the appearance of a diffuse electrophoretically slower migrating band on the SDS-PAGE gel (Fig. 2b), whereas the average number of introduced glycans was further determined by MALDI-TOF mass spectrometry [21] (Fig. 2c). Following this strategy, we produced OVA neoglycoconjugates displaying 2–3, 3–4, and 5–6 copies of N-glycan G0 per protein.

**Fig. 3:**
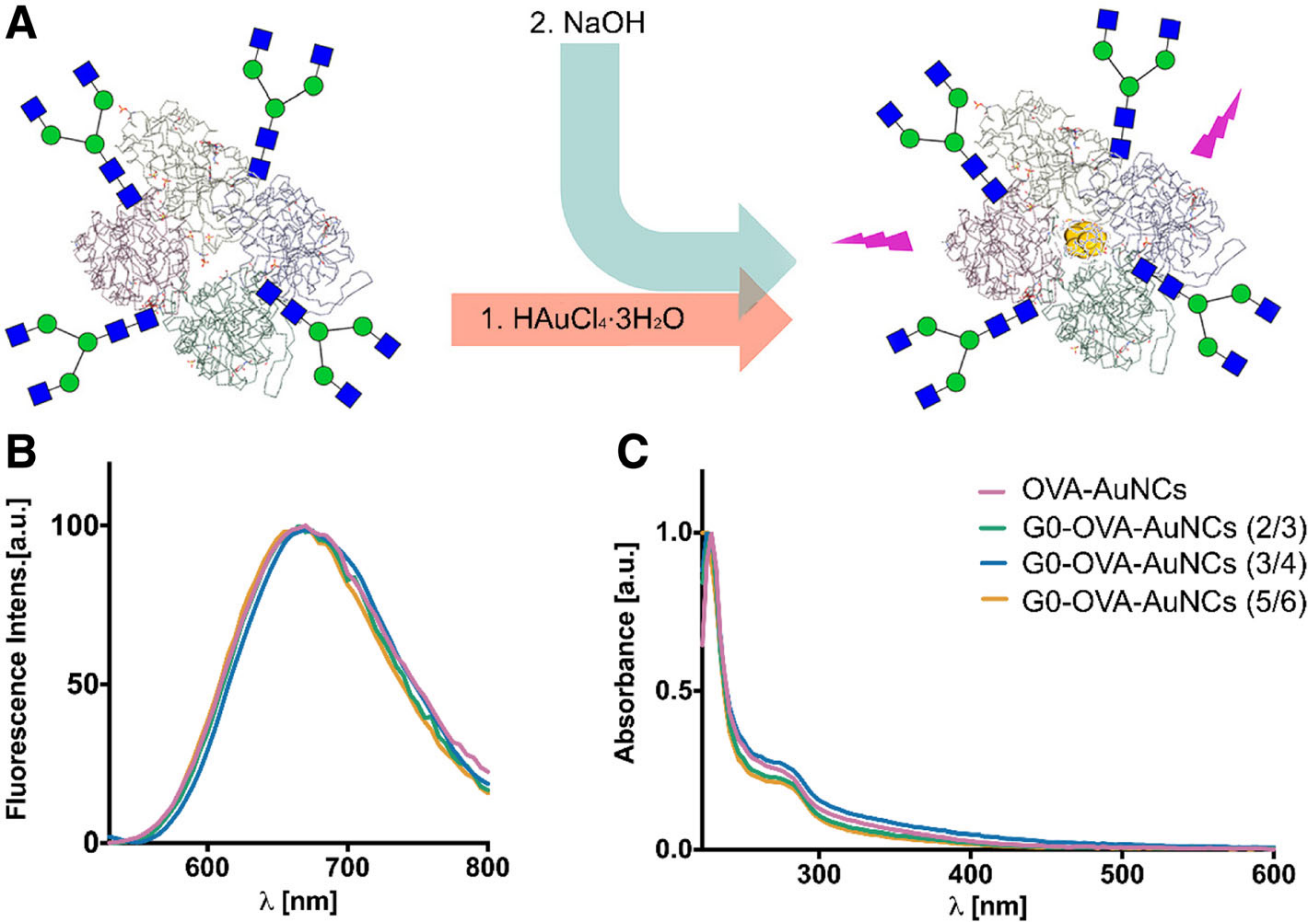
**a** Schematic representation of G0 glycan-derived OVA-AuNCs synthesis. **b** Fluorescence emission spectra of G0-OVA-AuNCs (blue, orange and green lines) compared to OVA-AuNCs (pink line). **c** UV-visible spectra of G0-OVA-AuNC (blue, orange and green lines) and OVA-AuNC (pink line)

Next, we employed the synthetic neoglycoproteins in the preparation of AuNCs (Fig. 3a) under previously optimized conditions and investigated the effect of the glycan functionalization and its valency on the physical and optical properties of the clusters. As shown on Fig. 3, the absorption UV-Vis and fluorescence emission of G0-OVA-AuNCs were identical to OVA-AuNCs with characteristic absorbance at 278 nm and red fluorescence emission with maximum around 670 nm (Fig. 3b, c).

**Fig. 4:**
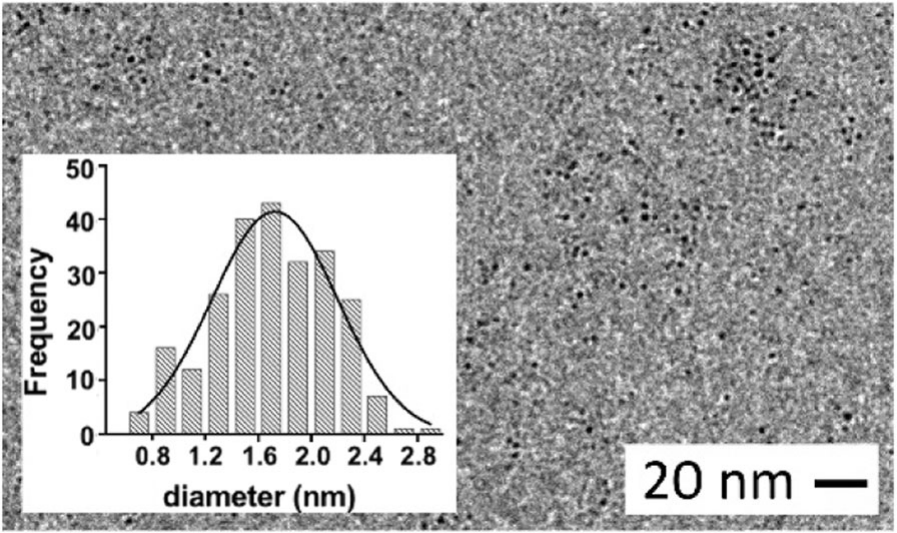
TEM image of G0-OVA-AuNCs (3/4). Insert: size distribution of gold core diameter of G0-OVA-AuNCs (3/4)

Furthermore, by TEM measurements, we established an average diameter of 1.6 ± 0.5 nm (Fig. 4) for G0-OVA-AuNCs core, which is comparable to the size of OVA-AuNCs (1.9 ± 0.7 nm). Thus, based on our results, we think that the glycans conjugated to OVA through lysine residues or the N-terminus do not affect the overall reductive potential of glycoproteins and further formation of clusters at the tested valencies [36]. Additionally, sugars seem not to hamper formation of cluster-stabilizing Au (I) thiolate polymers [37, 38] between the cysteine groups of protein and gold core, even when present in higher number (5–6 copies).

Finally, in the similar way as OVA-AuNCs, the secondary structure of the G0-OVA-AuNCs (3/4) protein was assigned by CD and revealed a random coil organization (Additional file 1: Figure S4). However, by the attachment of glycans to the protein scaffold of AuNCs, we introduce targeting molecules enabling interaction with carbohydrate-binding proteins and at the same time, an OVA protein remains only as a carrier which conformational changes will not alter the lectin recognition of the whole system. The antigenic capacity of denatured OVA in the production of antibodies in mice [39] and in T cell activation has been described. The activation of T cells is independent of the antigen secondary structure as it is mediated via recognition of certain amino acid sequences that are presented in both native and denatured OVA [40]. In fact, the antigen OVA 323-339 peptide account for the major specific T cell response to OVA and this peptide has been employed in nanoparticles to induce immune responses [41].

**Fig. 5:**
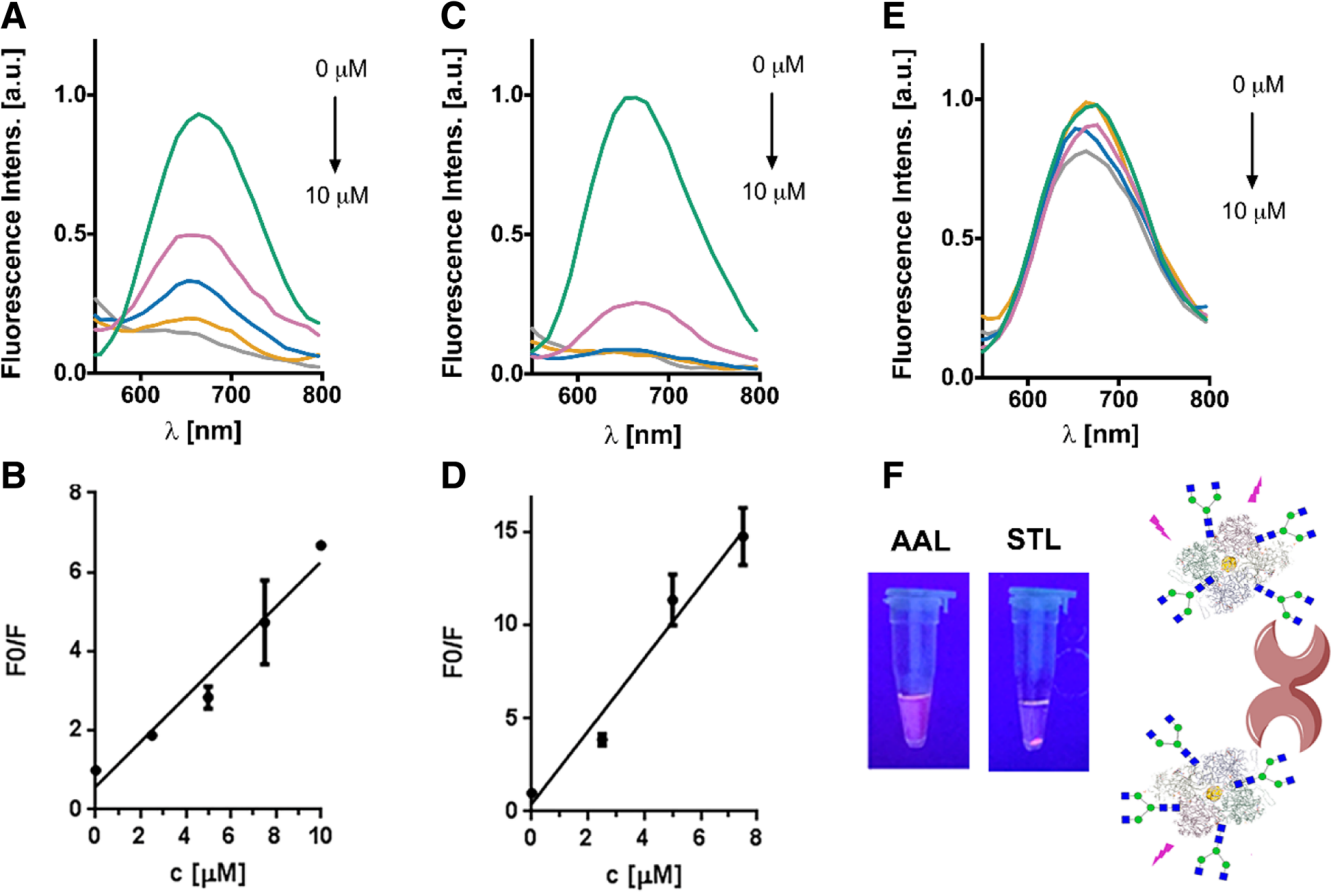
Lectin agglutination assays of G0-OVA-AuNCs (5/6). **a** Representative fluorescence spectra of the supernatants of OVA-G0 (5/6)-AuNCs solutions after incubation with BSL-II. **b** Representation of F0/F against increasing concentrations of BSL-II. **c** Representative fluorescence spectra of the supernatants of OVA-G0 (5/6)-AuNCs solutions after incubation with STL. **d** Representation of F0/F against increasing concentrations of STL. **e** Representative fluorescence spectra of the supernatants of OVA-G0 (5/6)-AuNCs after incubation with AAL. **f** Image corresponding to incubation of G0-OVA-AuNCs (5/6) with AAL and STL, under UV light illumination (365 nm). After incubation with STL, a visible precipitate was formed. On the right side: schematic illustration of binding between G0-OVA-AuNCs and plant lectin

To investigate the functionality of newly introduced glycan chains on neoglycoprotein protected gold nanoclusters, we examined their interaction with different plant lectins in agglutination experiments. We performed incubations of G0-OVA-AuNCs and OVA-AuNCs with two different plant lectins, Bandeiraea simplicifolia lectin-II (BSL-II) specific for terminal GlcNAc moieties and *Solanum tuberosum* lectin (STL), recognizing the core chitobiose present in the N-glycans [34, 42]. In addition and to discard any non-specific interactions between G0-OVA-AuNCs and lectins, *Aleuria aurantia* lectin (AAL) specific for L-fucose was included as a control. We added increasing concentrations of BSL-II, STL, and AAL lectins to a solution of G0-OVA-AuNCs (5/6). After incubation in the dark, the solutions were centrifuged and the fluorescence of the supernatant was measured. As demonstrated on Fig. 5, we could observe a decrease in fluorescence intensity in a concentration-dependent manner after incubation with BSL-II and STL (Fig. 5a–d), while the fluorescence intensity remained unchanged in the presence of AAL (Fig. 5e). After the incubation of G0-OVA-AuNCs (5/6) with STL, a visible precipitate under UV lamp irradiation was observed, whereas no precipitation appeared upon incubation with AAL (Fig. 5f). The relation of initial fluorescence values (F0) with the final values (F) after lectin incubation was represented against increasing concentrations of lectins (Fig. 5b, d). STL interaction with G0-OVA-AuNCs (5/6) showed linearity from 0 to 7.5 μM and the limit of detection was calculated based on the equation SD*3/S (where SD is the standard deviation of the calibration curve and *S* is the slope value) [12, 13] to be 2.35 μM (*y* = 1.95*x* + 0.4266, *R*^2^ = 0.97). In the same way, BSL-II interaction with G0-OVA-AuNCs (5/6) showed linearity from 0 to 10 μM and limit of detection (LOD) was calculated to be 2.83 μM (*y* = 0.5687*x* + 0.5838, *R*^2^ = 0.965). Interestingly, clusters containing a lower number of conjugated sugars like G0-OVA-AuNCs (2/3) (Additional file 1: Figure S7, A-B) did not show any significant change in fluorescence intensity upon addition of BSL-II and STL, indicating lack of agglutination. This behavior highlights an important effect of multivalent presentation of glycans on the cross-linking activity of lectins, even when only small differences in glycan density are applied. Importantly, no change in the fluorescence emission intensity of unconjugated OVA-AuNCs incubated with lectins was observed (Additional file 1: Figure S7, C-D). Even though native chicken OVA contains a single N-linked glycosylation site (Asn-292) predominantly substituted with high mannose or less abundant hybrid and complex type N-glycans [43], this sugar modification is not recognized by neither STL nor BSL-II, probably due to the monovalent presentation. This confirmed the presence of a specific carbohydrate-lectin interaction between chemically introduced G0 on G0-OVA-AuNCs and STL/BSL-II and ruled out non-specific binding of OVA-AuNCs to the lectins. We envisage a possible application of this system for the detection of carbohydrate binding proteins biomarkers. Nevertheless, the preparation of neoglycoproteins displaying a high number of glycan copies would increase the avidity of the construct toward the desired lectin improving the limit of detection [44].

We also studied the interaction of G0-OVA-AuNCs (5/6) with STL in the presence of cellular growth media to discriminate the possible effect of media components in carbohydrate protein interactions. G0-OVA-AuNCs (5/6) were dissolved in complete Iscove’s modified Dulbecco’s medium (IMDM) supplemented with fetal calf serum and incubated with increasing amounts of STL; the resulting solutions were analyzed by agarose gel electrophoresis. (Additional file 1: Figure S8). The electrophoretic mobility of G0-OVA-AuNCs (5/6) is maintained both in water and in complex medium. Nevertheless, in the presence of increasing amounts of STL, there was a dose-dependent displacement of G0-OVA-AuNCs (5/6) to the negative pole highlighting the protein carbohydrate interaction even in the presence of complex cellular media. This indicates that protein carbohydrate interactions with G0-OVA-AuNCs (5/6) are not impeded by the presence of media components.

**Fig. 6:**
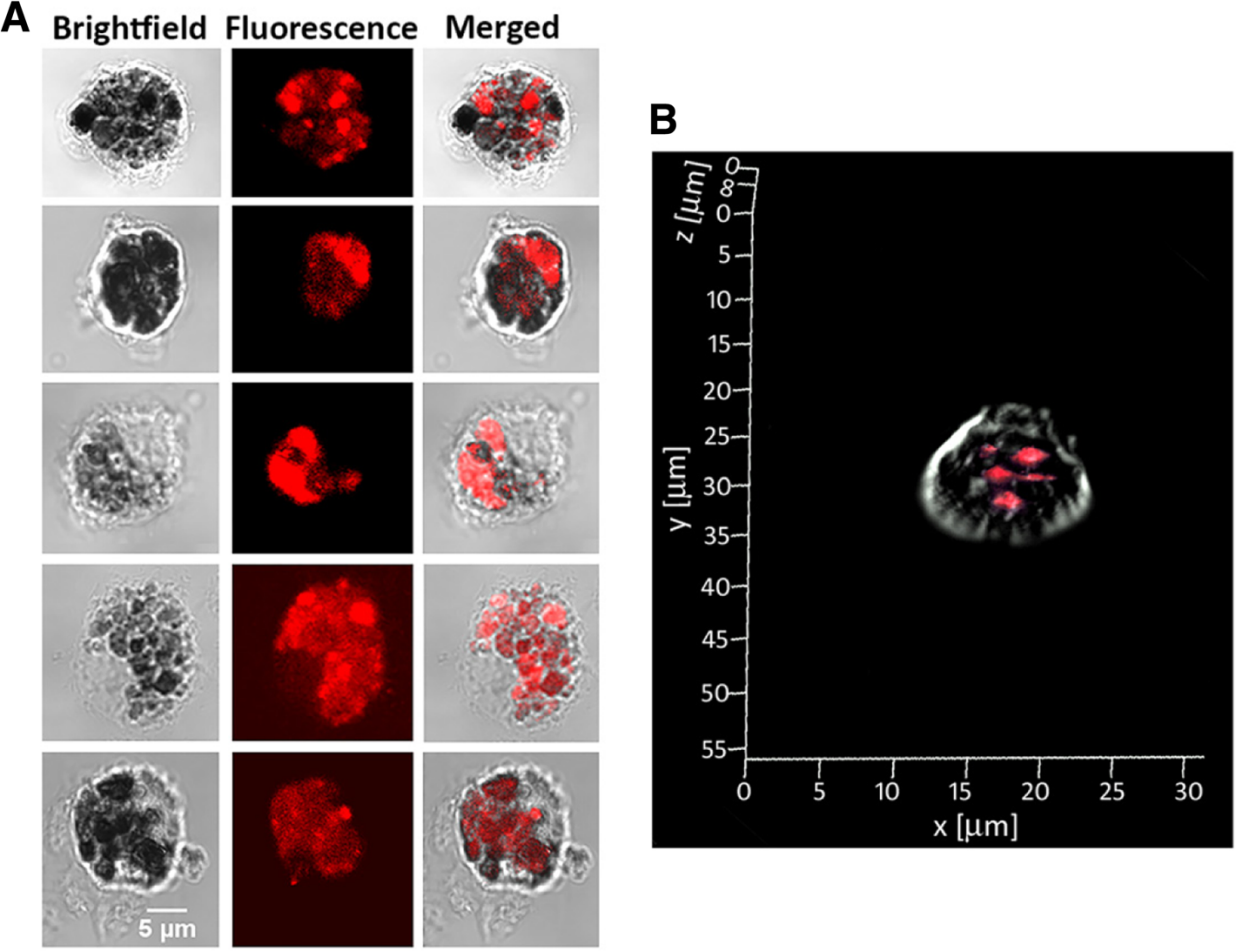
Uptake of G0-OVA-AuNCs (3/4) by murine DCs measured by confocal microscopy. **a** Representative images of CD11c^+^ cells after incubation with G0-OVA-AuNCs (3/4). **b** Z-stack image of a single dendritic cell representing uptake of G0-OVA-AuNCs inside the cell

The potential of clusters for fluorescence imaging and previous success with DCs targeting by G0-OVA neoglycoproteins [21] encouraged us to study G0-OVA-AuNCs in the uptake by murine DCs in vitro. We employed confocal fluorescence microscopy to visualize the internalization of self-fluorescent G0-OVA-AuNCs (3/4). Splenic DCs were isolated from C57BL/6J mice and CD11c^+^ population was purified by magnetic-activated cell sorting (MACS) (Additional file 1: Figure S9) and seeded on poly-d-lysine-coated glass cover-slips overnight. As a proof of concept, purified DCs were incubated with G0-OVA-AuNCs (3/4), washed to remove unbound materials, and fixed. After 40 min of incubation, confocal fluorescence microscopy images were acquired (Fig. 6) and we observed strong fluorescence for dendritic cells incubated with G0-OVA-AuNCs (3/4) demonstrating their effective internalization (Fig. 6a) and lack of fluorescence in the negative control (unstimulated CD11^+^ cells; Additional file 1: Figure S9). The internalization of nanoclusters was further confirmed by stacking of single DC images taken along their *Z*-axis (z-stack) and by reconstruction of a 3D image of a single DC to visualize fluorescence emission coming from inside the cell (Fig. 6b and Additional file 1: Figure S10).

In summary, we have prepared and characterized neoglycoprotein-protected gold nanoclusters that have been employed in agglutination experiments and in DCs uptake assay. On the example of specific plant lectin sensing, we have demonstrated the utility of G0-OVA-AuNCs for the analysis of carbohydrate-protein interactions. We also showed in vitro imaging of dendritic cells by fluorescence microscopy. However, further experiments have to be undertaken to first better understand the role of glycan in the uptake of nanoclusters by DCs (e.g., by following the localization of nanoclusters in the cellular compartments) and second, to study their bio- and cytocompatibility. Based on our previous results describing targeting properties of G0 glycan and increase in G0-OVA uptake by DCs compare to unconjugated OVA, we believe that G0-OVA-AuNCs could become an attractive alternative to fluorescently labeled neoglycoproteins.

## Conclusions

In conclusion, in this initial study, we present the first synthesis of neoglycoprotein-protected gold nanoclusters and evaluation of their physical and optical properties compared to unconjugated protein clusters. We confirmed the accessibility of sugar G0 on AuNCs in agglutination assays by specific interactions with plant lectins, which additionally highlighted the importance of a minimal glycan density for effective cross-linking activity of lectins. As a proof of concept, we also demonstrated the suitability of G0-OVA-AuNCs in the imaging of model murine DCs.

We believe that self-fluorescent neoglycoprotein functionalized AuNCs could become attractive tools enabling analysis and applications of carbohydrate-protein interactions. Based on their unique physical and chemical properties, such as large Stokes shifts, water solubility, or pH stability, G0-OVA-AuNCs could become an alternative to organic dye labels and be used for DCs visualization, carbohydrate-mediated uptake studies, and plant lectin sensing. Finally, by employing immunogenic carrier like OVA for the attachment of glycans, neoglycoprotein functionalized gold nanoclusters could not only allow for in-depth studies of antigen uptake, processing, and presentation, but also in the future, may find an application as fluorescent therapeutics or adjuvant molecules.

## Methods/Experimental

### Materials

All solutions were prepared in nanopure water (18 MΩ cm) from a Diamond UV water purification system (Branstead International, IA, Madrid, Spain). All glassware employed in the preparation of AuNCs was previously washed with aqua regia solution (HNO_3_: HCl, 1:3, *v*/*v*) and extensively rinsed with nanopure water. Gold (III) chloride trihydrate, sodium hydroxide, and triethylamine were purchased from Sigma Aldrich. LPS-free ovalbumin was purchased from Hyglos (Bernried, Germany). Disuccinimidyl suberate (DSS) and DMSO were purchased from Thermo Fisher Scientific. N-glycan G0 was chemically synthesized as previously described [21].

### AuNCs Synthesis. General Procedure

To a stirred solution of OVA or neoglycoprotein (15 mg mL^−1^) in nanopure water, an aqueous 0.1 M solution of HAuCl_4_·3H_2_O (4.2 mM, final concentration) was added dropwise. The resulting mixture was stirred at room temperature for 5 min and aqueous 1 M NaOH solution (150 mM final concentration) was added dropwise to increase the pH of the mixture. The resulting solutions were incubated at 100 °C for 6 min in Biotage® Initiator microwave reactor. Dialysis of AuNCs was performed on Slide-Z-lyser TM dialysis cassettes (10 K MWCO) from Thermo Fisher Scientific over nanopure water.

Fluorescence emission spectra of synthesized OVA and neoglycoprotein-protected AuNCs were measured using Nunc™ 96-Well Polystyrene Black plates, on Varioskan Flashmicroplate reader (Thermo Scientific) with excitation wavelength at λ 350 nm operating with a ScanIt Software.

Agarose gel electrophoresis of OVA-AuNCs and OVA protein was performed in 0.75% agarose gel at 80 V for 30 min. Visualization of OVA-protected AuNCs was performed under UV light irradiation (365 nm) and OVA protein was stained with Coomassie Blue G-250.

TEM imaging was conducted on a JOEL JEM-2100F field emission TEM with an accelerating voltage of 200 kV. AuNCs solutions were drop-cast onto copper TEM grids coated with ultrathin carbon support (Ted Pella, Redding, USA). The average diameter of the gold nanocluster was quantified using ImageJ (Java).

### Incubation of G0-OVA-AuNCs (5/6) with Plant Lectins

Ten microliters of G0-OVA-AuNCs (5/6) [0.2 mg mL^−1^] in TSM buffer (20 mM Tris·HCl, 150 mM NaCl, 2 mM CaCl_2_, 2 mM MgCl_2_, pH = 7.4) were placed onto Nunc™ 384-well polystyrene black plate. Subsequently, 10 μL of *Aleuria aurantia* lectin (AAL), *Solanum tuberosum* lectin (STL), and Bandeiraea simplicifolia lectin-II (BSL-II) in TSM buffer were added resulting in final protein concentrations of 0, 2.5, 5, 7.5, 10 μM. The corresponding solutions were incubated overnight in the dark under gentle shaking. Protein solutions were centrifuged at 11,000 g for 1 h and the fluorescence emission spectra of the supernatants was recorded with a Varioskan Flash microplate reader (Thermo Scientific) with excitation wavelength at λ 350 nm. To observe the precipitation upon formation of AuNCs-lectin complexes, a control experiment was performed. Then, 10 μL of G0-OVA-AuNCs (5/6) [0.2 mg mL^−1^] was incubated for 1 h with 10 μL of AAL and STL [40 μM], followed by centrifugation at 11,000 g (1 h). Images were taken under UV light irradiation (365 nm).

### Isolation of Mouse Spleen Dendritic Cells

Murine dendritic cells used in all experiments were isolated from C57BL/6J mice bred by Charles River (CIC biomaGUNE, San Sebastian, Spain). Animal experimental protocols were approved by the animal ethics committee of CIC biomaGUNE and were conducted in accordance with the ARRIVE guidelines and Directives of the European Union on animal ethics and welfare. Mice were kept under conventional housing conditions (22 ± 2 °C, 55 ± 10% humidity, and 12-h day/night cycle) and fed on a standard diet ad libitum*.* Mice were anesthetized with 2.5% isoflurane in 100% O_2_ and euthanized by cervical dislocation.

Spleens were obtained from C57BL/6J mice (*n* = 3, female, 27–28 weeks old). To isolate splenocytes, spleens were flushed with Iscove’s modified Dulbecco’s medium (IMDM) supplemented with 2 mM l-glutamine, 100 U/mL penicillin, 100 μg/mL streptomycin, and 10% fetal calf serum (FCS; PAN Biotech). The cell suspension was kept cold and filtered through a 40 μm cell strainer to remove cell aggregates. After centrifugation (300 g, 5 min, 4 °C), cell pellets were resuspended in 5 mL of freshly prepared erythrocyte lysis buffer (10% 100 mM Tris pH 7.5 + 90% 160 mM NH_4_Cl), mixed gently, and incubated at RT for 2 min. Cells were washed twice in complete IMDM medium and centrifuged before resuspension in MACS buffer (PBS, 0.5% BSA, 2 mM EDTA). Dendritic cells (CD11c^+^ cells) were isolated from a suspension of C57BL/6 murine spleen cells by magnetic-activated cell sorting using CD11c^+^ MicroBeads (Miltenyi). Cells incubated with magnetic microbeads were loaded on a MACS column placed in a magnetic field. Unbound cells passed through the column, while remaining CD11c^+^ cells were washed with MACS buffer and eluted from the column. To increase DC purity, the CD11c^+^ cell purification was repeated. The cell suspension was centrifuged, resuspended in IMDM complete medium, and counted.

### Uptake of G0-OVA-AuNCs (3/4) by DCs

CD11c^+^ cells from C57BL/6J mice (2 × 10^6^ cells) were seeded on poly-d-lysine-coated glass cover-slips overnight. G0-OVA-AuNCs (3/4) were added to cells (150 μg mL^−1^) and incubated for 40 min at 37 °C. After incubation, cells were carefully washed with cooled PBS and fixed with 3% paraformaldehyde at RT for 20 min. After washing with PBS and water, the cover-slips were mounted on slides using Vectashield® mounting medium. Fluorescent images were taken using the Zeiss LSM 510 laser scanning confocal microscope (Carl Zeiss) equipped with a UV laser (365 nm) and × 63 oil immersion objective.

## Additional File

Additional file 1: Electronic Supplementary Information (ESI) file containing experimental details for the preparation and characterisation of OVA-AuNCs (DLS, XPS, CD); a pH-stability and solubility study of OVA-AuNCs, synthesis of neoglycoproteins, analysis of lectin carbohydrate interactions by agarose gel electrophoresis and purification of CD11c+ DCs. (DOCX 4310 kb)

## Abbreviations

AAL: *Aleuria aurantia* lectin
AuNCs: Gold nanoclusters
BSA: Bovine serum albumin
BSL-II: Bandeiraea simplicifolia lectin-II
CD: Circular dichroism
CLRs: C-type lectin receptors
DCs: Dendritic cells
DMSO: Dimethyl sulfoxide
DSS: Disuccinimidyl suberate ester
FBS: Fetal bovine serum
G0: Biantennary N-glycan
G0-OVA-AuNCs: Neoglycoprotein functionalized gold nanoclusters
HAuCl_4_^.^_3_H_2_O: Gold tetrachloroauric (III) acid
IMDM: Iscove’s modified Dulbecco’s medium
IR: Infrared
LOD: Limit of detection
MACS: Magnetic-activated cell separation
OVA: Ovalbumin
QY: Quantum yield
STL: *Solanum tuberosum* lectin
TEM: Transmission electron microscopy
XPS: X-ray photoelectron spectroscopy

## References

[1] Lu Y, Chen W (2012). Sub-nanometre sized metal clusters: from synthetic challenges to the unique property discoveries. Chem Soc Rev.

[2] Kaur N, Nur Aditya R, Singh A, Kuo T-R (2018) Biomedical applications for gold nanoclusters: recent developments and future perspectives. Nanoscale Res Lett 13:302–314 10.1186/s11671-018-2725-9 PMC6158143 30259230

[3] Dreaden Erik C., Alkilany Alaaldin M., Huang Xiaohua, Murphy Catherine J., El-Sayed Mostafa A. (2012). The golden age: gold nanoparticles for biomedicine. Chem. Soc. Rev..

[4] Zhang Libing, Wang Erkang (2014). Metal nanoclusters: New fluorescent probes for sensors and bioimaging. Nano Today.

[5] Resch-Genger U, Grabolle M, Cavaliere-Jaricot S, Nitschke R, Nann T (2008). Quantum dots versus organic dyes as fluorescent labels. Nat Methods.

[6] Lavis Luke D. (2017). Teaching Old Dyes New Tricks: Biological Probes Built from Fluoresceins and Rhodamines. Annual Review of Biochemistry.

[7] Panchuk-Voloshina N, Haugland RP, Bishop-Stewart J, Bhalgat MK, Millard PJ, Mao F, Leung WY, Haugland RP (1999) Alexa Dyes, A series of new fluorescent dyes that yield exceptionally bright, Photostable Conjugates J Histochem Cytochem ;47:1179–1188 10.1177/002215549904700910 10449539

[8] Demchenko PA (2010). Collective effects influencing fluorescence emission in advanced fluorescence reporters in chemistry and biology II, Springer Series on Fluorescence.

[9] Wang Feng, Tan Wee Beng, Zhang Yong, Fan Xianping, Wang Minquan (2005). Luminescent nanomaterials for biological labelling. Nanotechnology.

[10] Kong Y, Chen J, Gao F, Brydson R, Johnson B, Heath G, Zhang Y, Wu L, Zhou D (2013). Near-infrared fluorescent ribonuclease-A-encapsulated gold nanoclusters: preparation, characterization, cancer targeting and imaging. Nanoscale.

[11] Xie J, Zheng Y, Ying JY (2009). Protein-directed synthesis of highly fluorescent gold nanoclusters. J Am Chem Soc.

[12] Govindaraju S, Ankireddy SR, Viswanath B, Kim J, Yun K (2017) Fluorescent gold nanoclusters for selective detection of dopamine in cerebrospinal fluid. Sci Rep 7:40298–40310 10.1038/srep40298 PMC5220289 28067307

[13] Chevrier Daniel M. (2012). Properties and applications of protein-stabilized fluorescent gold nanoclusters: short review. Journal of Nanophotonics.

[14] Dickson J, Geckler KE (2014). Synthesis of highly fluorescent gold nanoclusters using egg white proteins. Colloids Surf B Biointerfaces.

[15] Monsigny M, Roche AC, Duverger É, Srinivas O (2007). Neoglycoproteins. Comprehensive glycoscience. Chemistry to systems biology.

[16] Poole Jessica, Day Christopher J., von Itzstein Mark, Paton James C., Jennings Michael P. (2018). Glycointeractions in bacterial pathogenesis. Nature Reviews Microbiology.

[17] Varki A (2009) Essentials of Glycobiology 2nd edition, Part IV Glycan-binding Proteins, Cold Spring Harbor. p. 1–19

[18] Ogura A, Kurbangalieva A, Tanaka K (2016). Exploring the glycan interaction in vivo: future prospects of neo-glycoproteins for diagnostics. Glycobiology.

[19] Peumans WJ, van Damme EJ (1995). Lectins as plant defense proteins. Plant Physiol.

[20] Brown GD, Willment JA, Whitehead L (2018). C-type lectins in immunity and homeostasis. Nat Rev Immunol.

[21] Brzezicka K, Vogel U, Serna S, Johannssen T, Lepenies B, Reichardt NC (2016). Influence of Core β-1,2-Xylosylation on glycoprotein recognition by murine C-type lectin receptors and its impact on dendritic cell targeting. ACS Chem Biol.

[22] Johannssen Timo, Lepenies Bernd (2017). Glycan-Based Cell Targeting To Modulate Immune Responses. Trends in Biotechnology.

[23] Geijtenbeek Teunis B. H., Gringhuis Sonja I. (2016). C-type lectin receptors in the control of T helper cell differentiation. Nature Reviews Immunology.

[24] Streng-Ouwehand I, Ho NI, Litjens M, Kalay H, Boks MA, Cornelissen LA, Singh SK, Saeland E, Garcia-Vallejo JJ, Ossendorp FA, Unger WJU, van Kooyk Y (2016) Glycan modification of antigen alters its intracellular routing in dendritic cells, promoting priming of T cells. eLife ;5:e11765 10.7554/eLife.11765 PMC4811763 26999763

[25] Yoshimoto Junya, Tanaka Naoki, Inada Mitsuru, Arakawa Ryuichi, Kawasaki Hideya (2014). Microwave-assisted Synthesis of Near-infrared-luminescent Ovalbumin-protected Gold Nanoparticles as a Luminescent Glucose Sensor. Chemistry Letters.

[26] Wang LL, Qiao J, Liu HH, Hao J, Qi L, Zhou X, Li D, Nie Z, Mao L (2014). Ratiometric fluorescent probe based on gold nanoclusters and alizarin red-Boronic acid for monitoring glucose in brain microdialysate. Anal Chem.

[27] Qiao J, Mu X, Qi L, Deng J, Mao L (2013). Folic acid-functionalized fluorescent gold nanoclusters with polymers as linkers for cancer cell imaging. Chem Comm.

[28] Erickson HP (2009). Size and shape of protein molecules at the nanometer level determined by sedimentation, gel filtration, and electron microscopy. Biol Proced Online.

[29] Magde D, Wong R, Seybold PG (2002). Fluorescence quantum yields and their relation to lifetimes of rhodamine 6G and fluorescein in nine solvents: improved absolute standards for quantum yields. Photochem Photobiol.

[30] Kitagawa H, Kojima N, Nakajima TJ (1991). Studies of mixed-valence states in three-dimensional halogen-bridged gold compounds, Cs_2_Au^I^Au^III^X_6_, (X = Cl, Br or I). Part 2. X-Ray photoelectron spectroscopic study. J Chem Soc Dalton Trans.

[31] Greenfield NJ (2006). Using circular dichroism spectra to estimate protein secondary structure. Nat Protoc.

[32] Prasad Hari, Rao Rajini (2018). Histone deacetylase–mediated regulation of endolysosomal pH. Journal of Biological Chemistry.

[33] Sorkin Alexander, von Zastrow Mark (2002). Signal transduction and endocytosis: close encounters of many kinds. Nature Reviews Molecular Cell Biology.

[34] Serna S, Etxebarria J, Ruiz N, Martin-Lomas M, Reichardt NC (2010). Construction of *N*-glycan microarrays by using modular synthesis and on-Chip nanoscale enzymatic glycosylation. Chem Eur J.

[35] Eriksson M, Serna S, Maglinao M, Schlegel MK, Seeberger PH, Reichardt NC, Lepenies B (2014). Biological evaluation of multivalent Lewis X-MGL-1 interactions. Chembiochem.

[36] Xu Y, Sherwood J, Qin Y, Crowley D, Bonizzonic M, Bao Y (2014). The role of protein characteristics in the formation and fluorescence of au nanoclusters. Nanoscale.

[37] Söptei Balázs, Naszályi Nagy Lívia, Baranyai Péter, Szabó Ildikó, Mező Gábor, Hudecz Ferenc, Bóta Attila (2013). On the selection and design of proteins and peptide derivatives for the production of photoluminescent, red-emitting gold quantum clusters. Gold Bulletin.

[38] Pourceau Gwladys, Valle-Carrandi Lourdes del, Di Gianvincenzo Paolo, Michelena Olatz, Penadés Soledad (2014). On the chiroptical properties of Au(i)–thiolate glycoconjugate precursors and their influence on sugar-protected gold nanoparticles (glyconanoparticles). RSC Adv..

[39] Koch C, Jensen SS, Øster A, Houen G (1996). A comparison of the immunogenicity of the native and denatured forms of a protein. APMIS.

[40] Endres RO, Grey HM (1980). Antigen recognition by T cells. I. Suppressor T cells fail to recognize cross-reactivity between native and denatured ovalbumin. J Immunol.

[41] Safari D, Marradi M, Chiodo F, Th Dekker HA, Shan Y, Adamo R, Oscarson S, Rijkers GT, Lahmann M, Kamerling JP, Penadés S, Snippe H (2012). Gold nanoparticles as carriers for a synthetic Streptococcus pneumoniae type 14 conjugate vaccine. Nanomedicine.

[42] Pramod SN, Venkatesh YP, Mahesh PA (2007). Potato lectin activates basophils and mast cells of atopic subjects by its interaction with Core Chitobiose of cell-bound non-specific immunoglobulin E. Clin Exp Immunol.

[43] Harvey DJ, Wing DR, Küster B, Wilson IBH (2000). Composition of N-linked carbohydrates from ovalbumin and co-purified glycoproteins. J Am Soc Mass Spectrom.

[44] Laaf Dominic, Bojarová Pavla, Pelantová Helena, Křen Vladimír, Elling Lothar (2017). Tailored Multivalent Neo-Glycoproteins: Synthesis, Evaluation, and Application of a Library of Galectin-3-Binding Glycan Ligands. Bioconjugate Chemistry.

