# Supplementary material for: Fluorescent Neoglycoprotein Gold Nanoclusters: Synthesis and Applications in Plant Lectin Sensing and Cell Imaging
Source: Nanoscale Res Lett. 2018 Nov 12;13:360. doi: 10.1186/s11671-018-2772-2 (PMC6232188; doi:10.1186/s11671-018-2772-2)
Supplement: Supplementary file 1 — Electronic Supplementary Information (ESI) file containing experimental details for the preparation and characterisation of OVA-AuNCs (DLS, XPS, CD); a pH-stability and solubility study of OVA-AuNCs, synthesis of neoglycoproteins, analysis of lectin carbohydrate interactions by agarose gel electrophoresis and purification of CD11c+ DCs. (DOCX 4310 kb) [file 11671_2018_2772_MOESM1_ESM.docx]

**Additional file 1**

**Fluorescent Neoglycoprotein Gold Nanoclusters: Synthesis and Applications in Plant Lectin Sensing and Cell Imaging**

Katarzyna Brzezicka^a,b*^, Sonia Serna^a^ and Niels Christian Reichardt^a,c*^

1. Glycotechnology Laboratory, CIC biomaGUNE, Paseo Miramon 182, 20014 San Sebastian, Spain, ka.brzezicka@gmail.com
2. current address: Departments of Molecular Medicine and Microbiology and Immunology, The Scripps Research Institute, La Jolla, CA, 92037, USA, kbrzez@scripps.edu
3. CIBER-BBN, Paseo Miramon 182, 20014 San Sebastian, Spain, nreichardt@cicbiomagune.es

**Table of contents:**

1. Optimization of synthesis of OVA-AuNCs S1
2. Characterization of OVA-AuNCs S2-3
3. Stability of OVA-AuNCs at different pH range and in the presence of FBS S4
4. Solubility of OVA AuNCs in water and in complete medium S5
5. Synthesis and characterisation of neoglycoproteins S6
6. Incubation of G0-OVA-AuNCs (2/3) and OVA AuNCs with *Solanum tuberosum* lectin

(STL) and *Bandeiraea simplicifolia lectin II* (BSL-II) S7

1. Interaction of G0-OVA-AuNCs (5/6) with STL in complex cellular media S8
2. Scatter plots of MACS-purified DCs and uptake of G0-OVA-AuNCs by murine DCs S9
3. **Optimization of synthesis of OVA-AuNCs**

A solution of HAuCl_4_·3H_2_O (4.2 mM final concentration) was added to stirred solutions of OVA protein in water at different concentrations (15, 10, 5, 2.5, 1.25 and 0.65 mg·mL-1), the resulting mixtures were stirred at room temperature for five minutes and NaOH (150 mM final concentration) was added dropwise to increase the pH of the mixture. The resulting solutions were incubated at 100 ºC for 6 minutes in Biotage Initiator microwave reactor. Fluorescence emission spectra were measured using Nunc™ 96-Well Polystyrene Black plates, on Varioskan Flashmicroplate reader (Thermo Scientific) with excitation wavelength at λ 350 nm operating with a ScanIt Software.


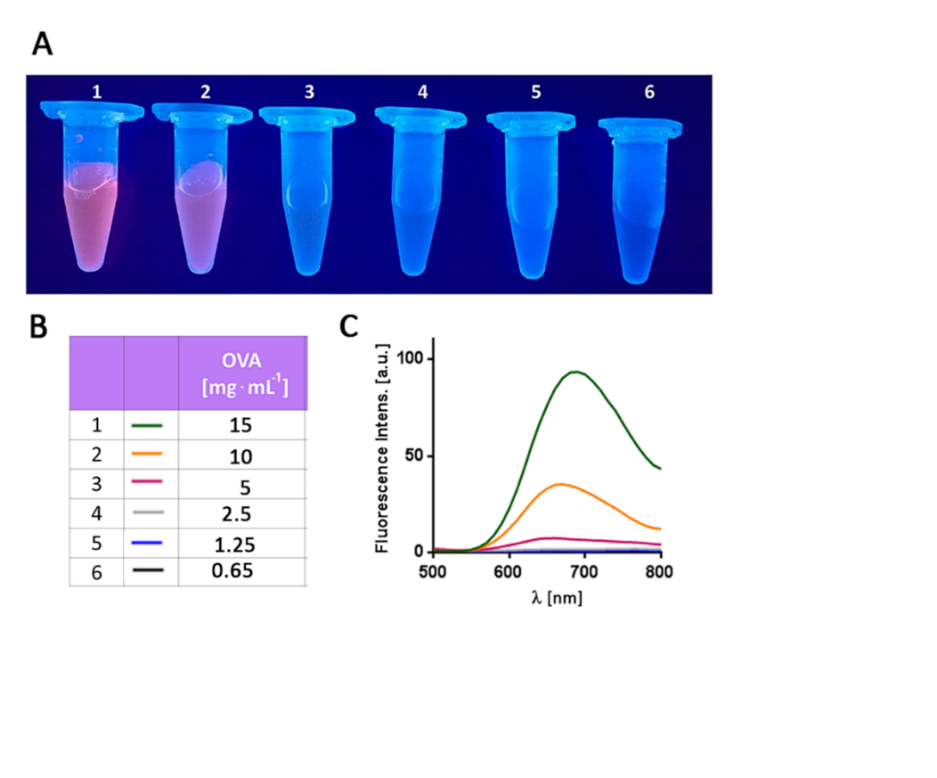


**Figure S1.** Optimization of the OVA-AuNCs synthesis; **A.** Image of OVA-AuNCs solutions (different protein concentrations) under UV light illumination (365 nm); **B.** Protein concentrations employed during the synthesis; **C.** Fluorescence emission spectra of OVA-AuNCs at six different concentrations of OVA used.

1. **Characterization of OVA-AuNCs:**

**
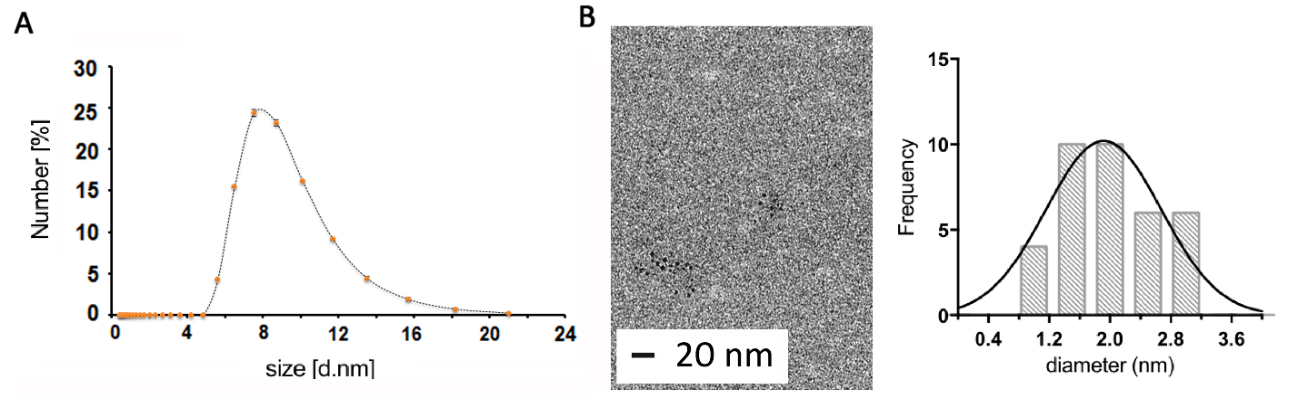
Dynamic light scattering (DLS)** was performed at fixed scattering angle of 90º on a Malvern Zetasizer Nano ZS. Before the measurements, to the cluster solution [5 mg^.^ mL^-1^], NaCl [0.1 M] was added.

**Figure S2.** **A.** DLS of OVA-AuNCs indicating the average hydrodynamic size of OVA-AuNCs; **B.** TEM image of OVA-AuNCs and the size distribution of gold core diameter.

**X-ray photoelectron spectroscopy (XPS)**: Analysis by XPS was performed in a SPECS SAGE HR 100 system spectrometer in an ultrahigh vacuum (UHV) chamber. The X-ray sources employed for this analysis were a non-monochromatic Mg Kα (1253.6 eV) and 250 W or Al Kα operated at 1.25 kV and 300W, calibrated using the 3d_5/2_ line of Ag with a full width at half maximum (FWHM) of 1.1 eV. The take- off angle was fixed at 90º and the analysis was conducted at a pressure of ~10-^6^ Pa. Surfaces were brought into the XPS chamber within 5 min after cleaning/preparation. The selected resolution for the spectra was 30 eV of Pass Energy and 0.5 eV/step for the general survey spectra and 15 eV of Pass Energy and 0.15 eV/step for the detailed spectra of the different elements. Spectra were analyzed with the CasaXPS 2.3.15dev87 software. The analysis consisted of satellite removal, Shirley background subtraction, calibration of the binding energies related to the C 1s C-C peak at 285 eV, and asymmetric peak fitting with Gaussian-Lorentzian line shapes where the FWHM of all the peaks were constrained while the peak positions and areas were set free. Samples are prepared by dehydration on clean titania substrates where titania is selected as a substrate to avoid any possible overlap of gold signal with signal of silica coming from wafer or glass. HAuCl_4_·3H_2_O salt is measured as received in custom made powder holder.


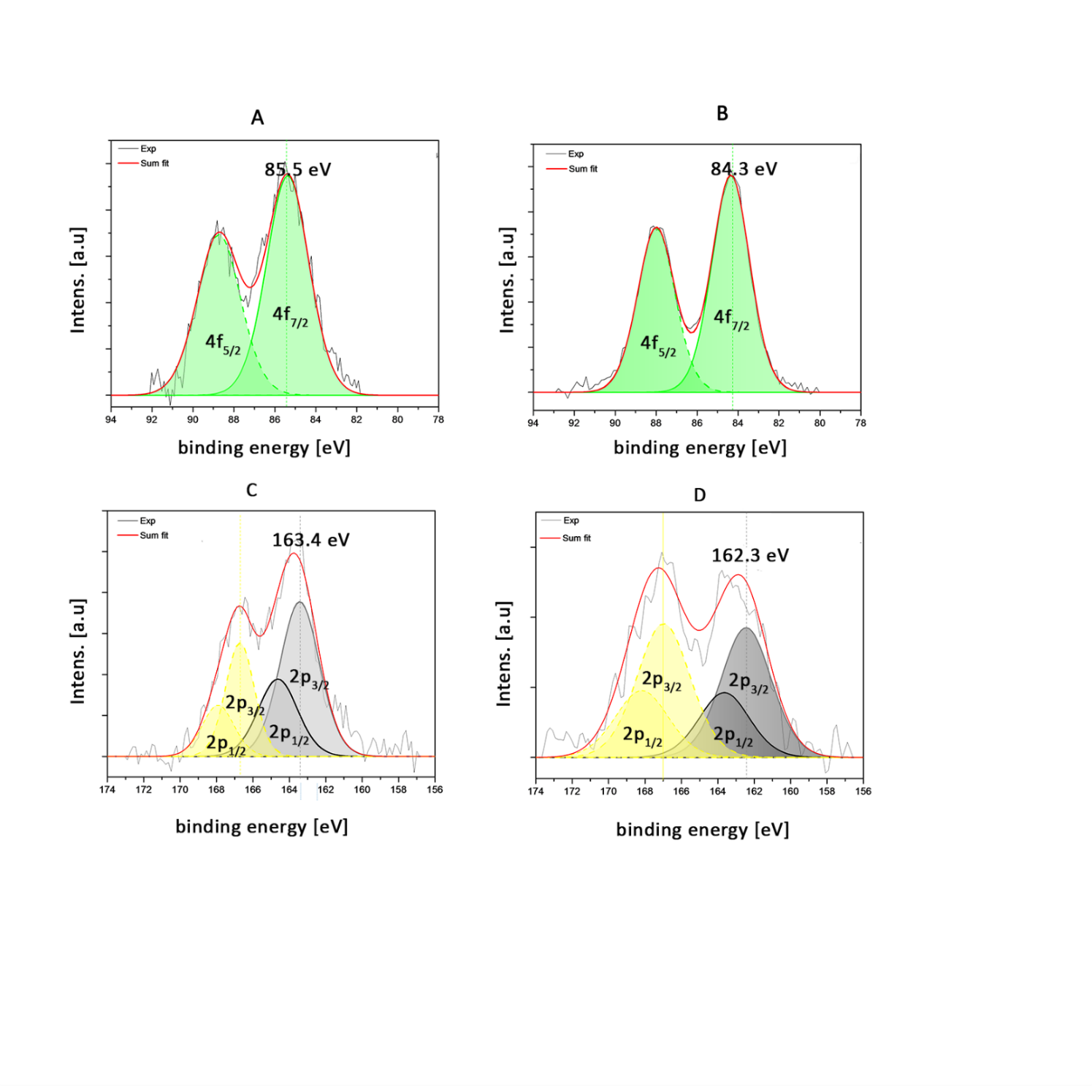


**Figure S3.** X-ray photoelectron spectra (XPS) of HAuCl_4_^.^ 3H_2_O, OVA solution and OVA-AuNCs **A.** Au 4f region for solid HAuCl_4_^.^ 3H_2_O **B.** Au 4f region of OVA-AuNCs solution **C.** S 2p region of OVA solution **D.** OVA-AuNCs solutions.

**Circular dichroism** (CD) spectra were recorded using Jasco J815 and 0.25 mg/mL protein or glycoprotein solutions in nanopure water.


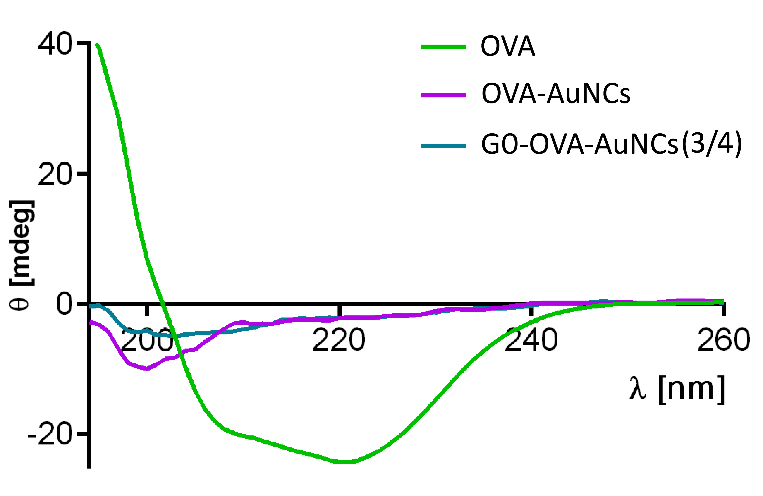


**Figure S4.** Circular dichroism (CD) spectra of OVA solution (green line), OVA-AuNCs (violet line) and G0-OVA-AuNCs (blue line) indicating conformational changes in the native OVA upon stabilization of OVA-AuNCs.

1. **Stability of OVA-AuNCs in different pH range and in the presence of FBS**

OVA-AuNCs were dissolved in aqueous solutions at pH values ranging from pH=3 to pH=11, the solutions were incubated for three hours at room temperature in the dark. After this time, fluorescence emission was recorded. The stability and solubility of OVA-AuNCs was evaluated in a solution of fetal bovine serum (FBS). Freeze-dried OVA-AuNCs were easily re-suspended in 10% solution of FBS in PBS buffer and left overnight at 37 °C. After this time, fluorescence emission was recorded.


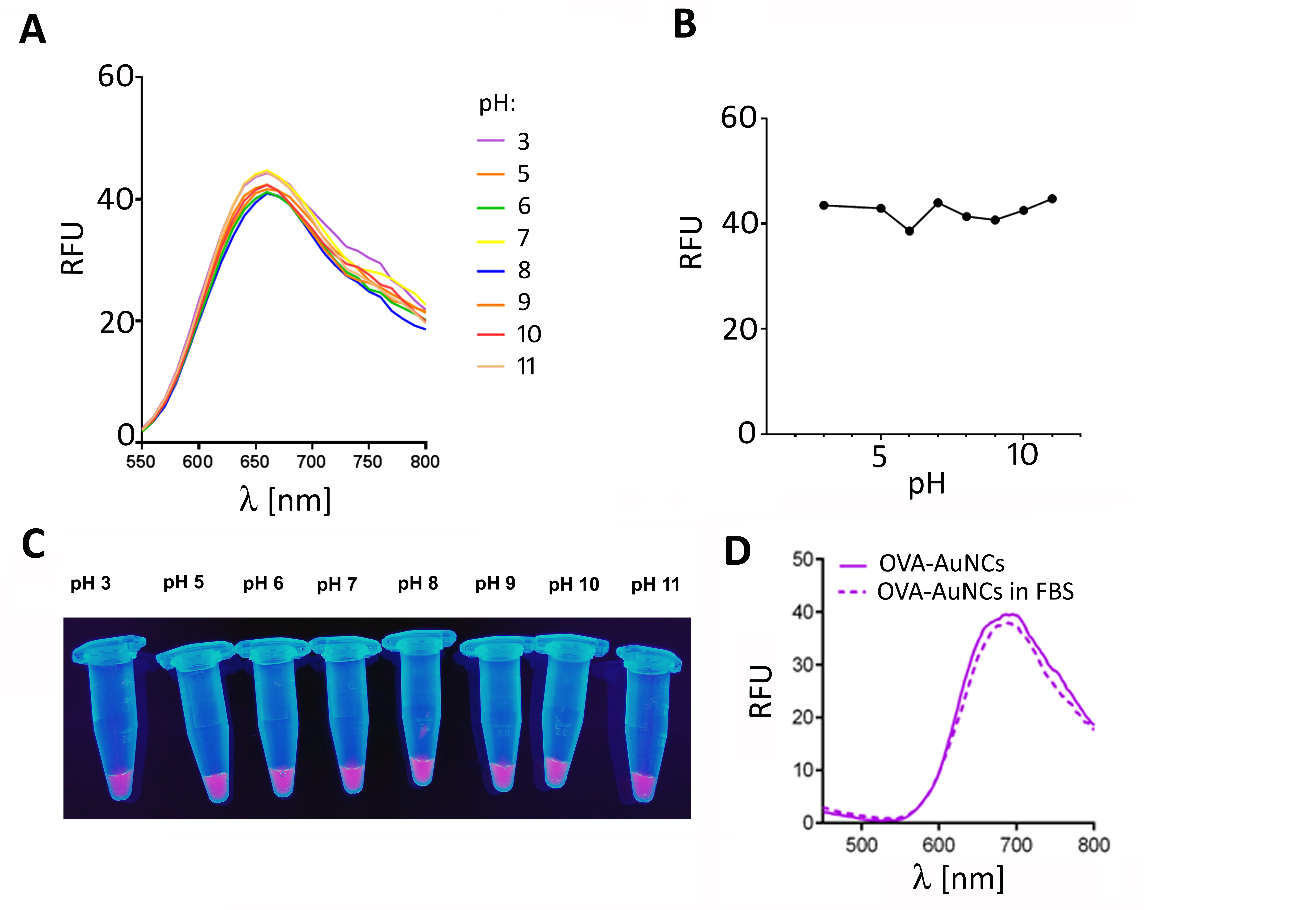


**Figure S5.** **A.** Fluorescence emission spectra of OVA-AuNCs incubated in solutions of pH range between 3-11; **B.** pH–response fluorescence curved derived from Panel A; **C** Image corresponding to incubation of OVA-AuNCs at different pH, under UV light illumination (365 nm); **D.** Fluorescence emission spectra of OVA-AuNCs (violet line) and OVA-AuNCs after incubation with 10% FBS (violet dashed line)

1. **Solubility of OVA AuNCs in water and in complete medium.**

OVA AuNCs were dissolved at 40 mg/mL concentration in nanopure water and in complete medium: Iscove's Modified Dulbecco's medium (IMDM) supplemented with 2 mM L-glutamine, 100 U/mL penicillin, 100 μg/mL streptomycin, and 10% fetal calf serum (FCS). Serial 1:2 dilutions were prepared (40 mg/mL to 0.3 mg/mL) and fluorescence emission was measured.


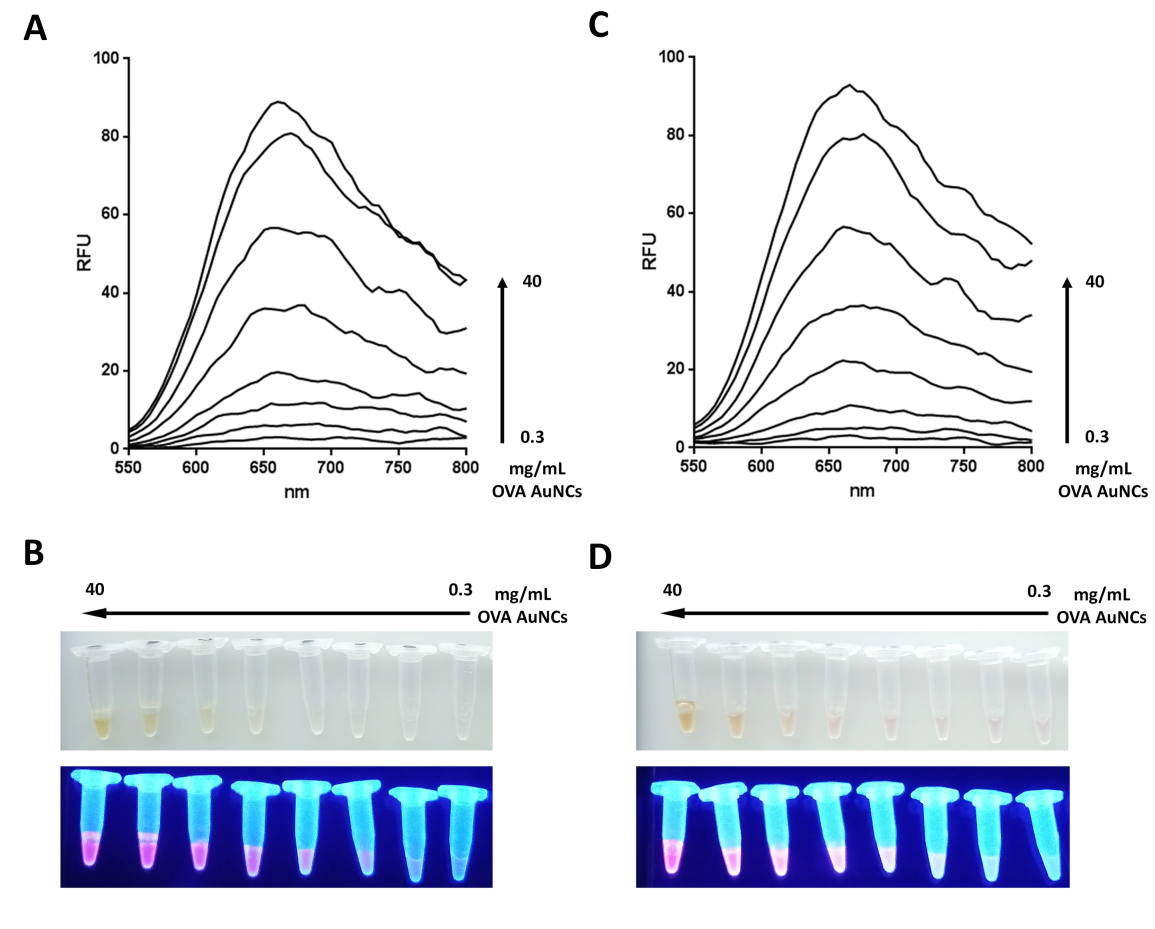


**Figure S6**. Solubility of OVA AuNCs in water and in IMDM medium. **A.** Fluorescence emission spectra of OVA AuNCs solutions in water. **B.** Images under visible light (up) and under UV irradiation (down) of OVA AuNCs solutions in water. **C.** Fluorescence emission spectra of OVA AuNCs solutions in complete medium. **D.** Images under visible light (up) and under UV irradiation (down) of OVA AuNCs solutions in complete medium. Arrows indicate increasing concentrations of OVA AuNCs solutions.

1. **Synthesis and characterisation of neoglycoproteins**

**General procedure:** N-glycan G0 was conjugated first to bifunctional DSS linker and after reacted with OVA protein solution. DSS linker (13 eq.) was dissolved in DMSO (20 mM) and activated with 20 µL of triethylamine. N-glycan G0 (1 eq.) was dissolved in DMSO and slowly added to the solution of linker. Reaction was left stirring at room temperature for 90 min. Conjugation product was extracted with PBS (1.5 mL) and washed 3 times with chloroform (centrifuge, 3000 g). In order to generate G0-OVA neoglycoproteins with different degree of substitution, different excess (40, 50 or 72.5 eq.) of N-glycan-DSS conjugate were combined with OVA and the resulting solutions were incubated at room temperature overnight. Neoglycoproteins were dialyzed against PBS. The neoglycoproteins were characterized by MALDI-TOF mass spectrometry and by sodium dodecyl sulfate polyacrylamide gel electrophoresis (SDS-PAGE). The use of 40 equivalents produced G0-OVA neoglycoprotein with 2/3 copies of sugar, the use of 50 equivalents produced G0-OVA with 3/4 copies of sugar and the use of 72.5 equivalents produced G0-OVA with 5/6 copies of sugar.

**MALDI-TOF MS** A small volume of neoglycoproteins and OVA solutions were subjected to buffer exchange against nanopure water using 10 kDa Amicon filter devices to avoid signal suppression due to the presence of salts. Protein solutions (1 µL) were deposited on a MTP 384 polished steel MALDI plate (Bruker Daltonics, Bremen, Germany) and dried at room temperature. Matrix solution (2 μL) containing sinapic acid, 4 mg/mL in CH3CN: aqueous TFA 1% (1:1) was deposited on top and dried at room temperature. MALDI‐TOF mass measurements were performed on an Ultraflextreme III time‐of‐flight mass spectrometer equipped with a pulsed Nd:YAG laser (λ 355 nm) and controlled by FlexControl 3.3 software (Bruker Daltonics, Bremen , Germany). The acquisitions (total of 2000-3000) were carried out in linear positive ion mode with pulsed ion extraction of 450 ns and laser frequency of 1000 Hz. Laser fluence was set up to 60-80 % and the m/z range was chosen according to the mass of the sample. The accumulated spectra were processed using the Bruker software FlexAnalysis 3.3.

Sodium dodecyl sulfate polyacrylamide gel electrophoresis (SDS-PAGE) was performed under reducing conditions. Protein sample buffer was prepared with 10% w/v SDS, 10 mM β-mercapto-ethanol, 20 % v/v glycerol, 0.2 M Tris·HCl pH 6.8 and 0.05% w/v bromophenol blue. Protein samples were heated in sample buffer for 5 min at 90 ̊C and loaded on 10% acrylamide gel. Electrophoresis was performed using Mini-Protein Tetra cell electrophoresis system (Bio-Rad) and gel was stained with Coomassie Brilliant G-250.

1. **Incubation of G0-OVA-AuNCs (2/3) and OVA AuNCs with *Solanum tuberosum* lectin (STL) and *Bandeiraea simplicifolia lectin II* (BSL-II)**

10 µL of each AuNCs [0.2 mg/mL] in TSM buffer (20 mM Tris·HCl, 150 mM NaCl, 2 mM CaCl_2_, 2 mM MgCl_2_, pH=7.4) were placed onto Nunc™ 384-well polystyrene black plate. Subsequently, 10 µL of *Solanum* *tuberosum* lectin (STL) and *Bandeiraea* *simplicifolia* lectin-II (BSL-II) in TSM buffer were added resulting in final protein concentrations of 0, 2.5, 5, 7.5, 10 µM. The corresponding solutions were incubated overnight in the dark under gentle shaking. Protein solutions were centrifuged at 11000 g for 1h and the fluorescence emission spectra of the supernatants was recorded with a Varioskan Flash microplate reader (Thermo Scientific) with excitation wavelength at λ 350 nm.


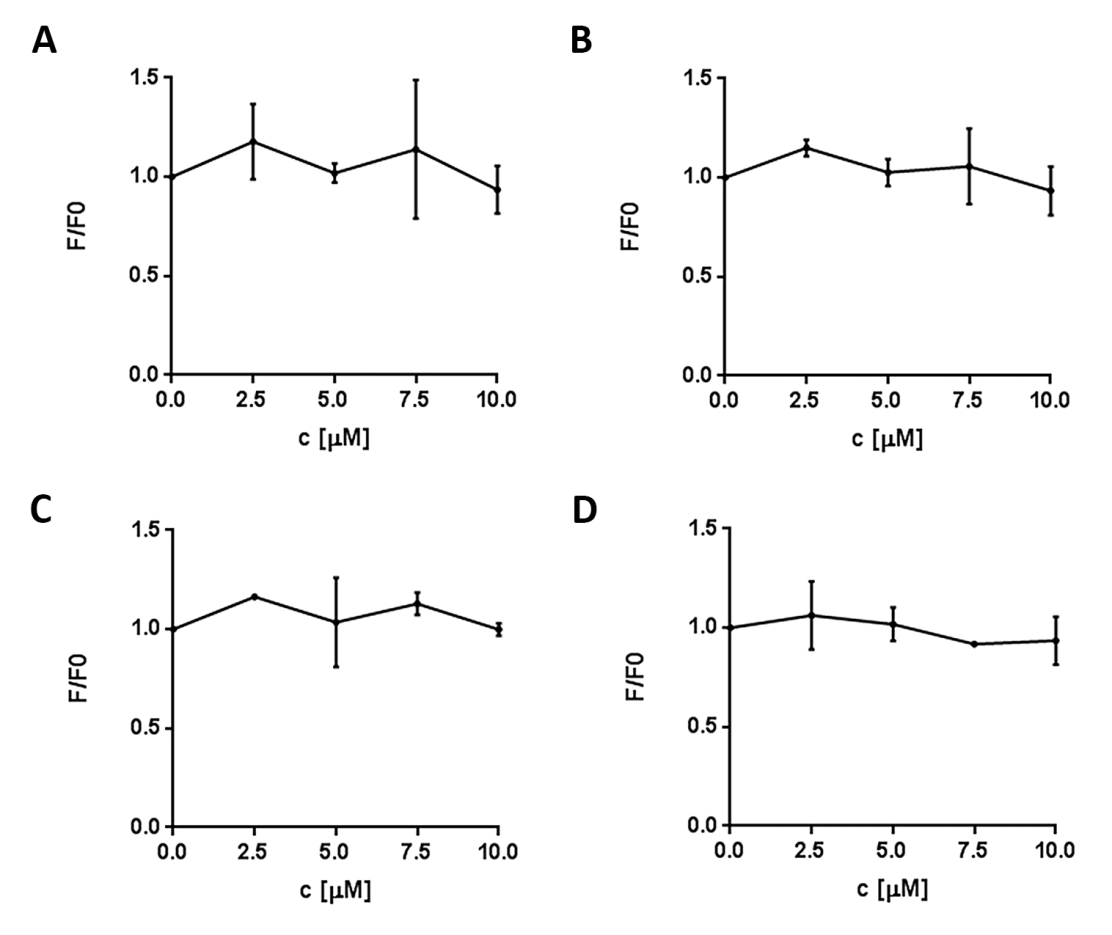


**Figure S7.** **A.** Incubation of G0-OVA-AuNCs (2/3) with *Solanum tuberosum* lectin (STL) **B.** Incubation of G0-OVA-AuNCs (2/3) with *Bandeiraea simplicifolia lectin II* (BSL-II). **C.** Incubation of OVA AuNCs with *Solanum tuberosum* lectin (STL) **D.** Incubation of OVA AuNCs with *Bandeiraea simplicifolia lectin II* (BSL-II)

The initial fluorescence F0 for each solution is maintained after the incubation with both lectins at different concentrations indicating lack of agglutination.

1. **Interaction of G0-OVA-AuNCs (5/6) with Solanum tuberosum lectin in complex cellular media.**

G0-OVA-AuNCs (5/6) were disolved in Iscove's Modified Dulbecco's medium (IMDM) supplemented with 2 mM L-glutamine, 100 U/mL penicillin, 100 μg/mL streptomycin, and 10% fetal calf serum (FCS) and incubated with increasing amounts of STL lectin in PBS (5, 10, 15, 20 μM). The resulting solutions were incubated at 37ºC for one hour and analysed by agarose gel electrophoresis.

Agarose gel electrophoresis was performed in 0.5 % agarose gel running in TAE buffer at 80 V for 60 minutes. G0-OVA-AuNCs (5/6) visualization was performed under UV light irradiation (365 nm) and proteins were stained with Coomassie Blue G-250.

Complete media for cell culture generally contains as major protein component bovine serum albumin (lane 1, Commassie blue). G0-OVA-AuNCs (5/6) both dissolved in water (lane 2) and in complex media (lane 3) showed similar electrophoretic mobility to the positive pole, indicating their negative charge. In the presence of STL (Figure S7, B) an important mobility shift to the negative pole of G0-OVA-AuNCs (5/6) was observed due to the interaction with STL protein (lanes 4 to 7, UV). On the other hand, BSA from media remains unaltered (Lanes 4 to 7, Commassie staining).


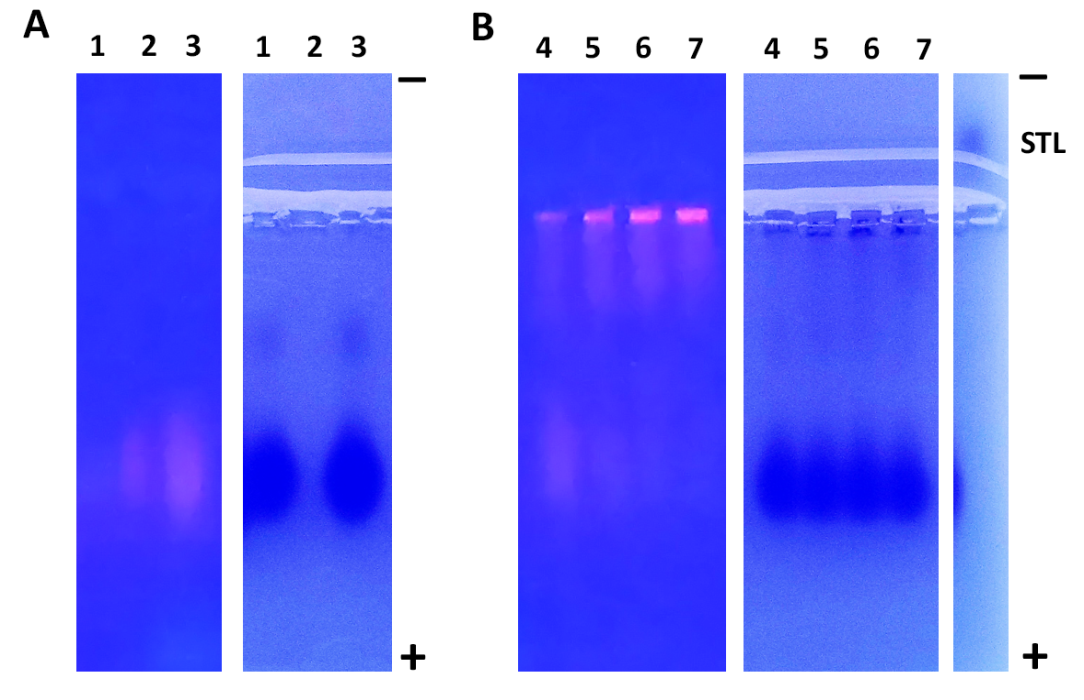


**Figure S8. A.** Agarose gel electrophoresis of G0-OVA-AuNCs (5/6).(Left: visualized under UV irradiation; Right: Commassie blue G-250 staining) 1. Complete IMDM medium containing 10% fetal calf serum. 2. G0-OVA-AuNCs (5/6) in water. 3. G0-OVA-AuNCs (5/6) in complete IMDM medium. **B.** Agarose gel electrophoresis of G0-OVA-AuNCs (5/6) after incubation with *Solanum tuberosum* lectin (STL). 4. STL (5 μM); 5. STL (10 μM); 6. STL (15 μM); 7. STL (20 μM).

1. **Scatter plots of MACS-purified DCs and uptake of G0-OVA-AuNCs by murine DCs**


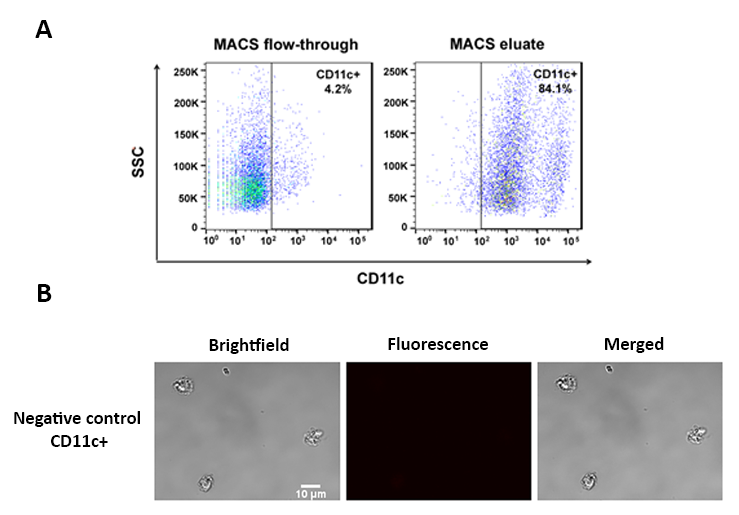


**Figure S9** A. Representative dot plots of MACS-purified DCs and MACS flow-through. Purity of the DC isolation was analysed by staining with an anti-CD11c antibody. B. Representative confocal image of unstimulated CD11c+ cells showing no fluorescence emission.

**Figure S10** Visualization of Z-stack images of two dendritic cell representing uptake of G0-OVA-AuNCs inside the cell.
